# Human papillomavirus (HPV) vaccine implementation in low and middle-income countries (LMICs): Health system experiences and prospects^☆^

**DOI:** 10.1016/j.vaccine.2013.06.016

**Published:** 2013-08-20

**Authors:** Jannah Wigle, Ernestina Coast, Deborah Watson-Jones

**Affiliations:** a Department of Social Policy, London School of Economics, Houghton Street, London WC2A 2AE, UK; b Clinical Research Department, London School of Hygiene & Tropical Medicine, Keppel Street, London WC1E 7HT, UK; c Mwanza Intervention Trials Unit, National Institute for Medical Research, Mwanza, Tanzania

**Keywords:** Human papillomavirus, HPV, Vaccine, Cervical cancer, Sexually transmitted infection, Low- and middle-income countries (LMICs)

## Abstract

Prophylactic vaccines for human papillomavirus (HPV) are being introduced in many countries for the prevention of cervical cancer, the second most important cause of cancer-related death in women globally. This is likely to have a significant impact on the future burden of cervical cancer, particularly where screening is non-existent or limited in scale. Previous research on the challenges of vaccinating girls with the HPV vaccine has focused on evidence from developed countries. We conducted a systematic search of the literature in order to describe the barriers and challenges to implementation of HPV vaccine in low- and middle-income countries. We identified literature published post-2006 to September 2012 from five major databases. We validated the findings of the literature review with evidence from qualitative key informant interviews. Three key barriers to HPV vaccine implementation were identified: sociocultural, health systems and political. A linked theme, the sustainability of HPV vaccines programmes in low- and middle-income countries, cuts across these three barriers. Delivering HPV vaccine successfully will require multiple barriers to be addressed. Earlier research in developed countries emphasised sociocultural issues as the most significant barriers for vaccine roll-out. Our evidence suggests that the range of challenges for poorer countries is significantly greater, not least the challenge of reaching girls for three doses in settings where school attendance is low and/or irregular. Financial and political barriers to HPV vaccine roll-out continue to be significant for many poorer countries. Several demonstration and pilot projects have achieved high rates of acceptability and coverage and lessons learned should be documented and shared.

## Highlights

• Multi-method review of challenges introducing HPV vaccine in developing countries.
• HPV vaccine delivery methods can overcome inadequate health systems.
• Political will is essential for successful HPV vaccine roll-out.

## 1 Introduction

Prophylactic vaccines for human papillomavirus (HPV) prevent cervical cancer, the second major cause of cancer-related deaths amongst women in less developed countries [1,2]. Cervical cancer causes approximately 275,000 deaths annually worldwide, of which 88% occur in low income countries (LICs) [1,3] Implementation of a primary prevention vaccine is likely to have a significant impact on the burden of cervical cancer, particularly where screening is non-existent or limited in scale or of poor quality [4].

HPV is the most common sexually transmitted infection (STI) globally, prevalent in approximately 11–12% of women and is the major cause of cervical cancer [1,3]. There are over 100 types of HPV genotypes, with HPV types 16 and 18 being responsible for approximately 70% of cervical cancers, and types 6 and 11 for 96–100% of genital warts infections [3]. The relationship between cervical cancer and HPV is often poorly recognised by both policymakers and women [5].

There are two commercially available prophylactic HPV vaccines [6,7]. At least 110 countries have licensed the bivalent HPV vaccine (Cervarix™) that protects against HPV genotypes 16 and 18 and over 120 countries have licensed the quadrivalent vaccine (Gardasil™) that protects against HPV genotypes 6, 11, 16 and 18 [8]. However, licensure does not mean that all these countries are currently providing the vaccine within the public sector. The inclusion of the HPV vaccine into national immunisation programmes has varied widely; in 2012 of the 51 countries implementing national HPV vaccination programmes, only six were LMICs. The number of smaller-scale or demonstration projects continues to increase. Currently 26 low-, middle- and upper-middle income countries are engaged in piloting activities to test delivery strategies, understand and overcome potential barriers and to inform decision-making for future national roll-out of the vaccine [8–11].

The primary target group for HPV vaccination is girls aged 9–13 years in order to vaccinate prior to the onset of sexual activity and therefore before HPV is acquired, [12]. Although many Latin America and Caribbean, African countries and Asian LIC have been able to reach adolescent girls with single or multiple dose vaccines such as Hepatitis B, measles or tetanus toxoid to prevent neonatal tetanus [13–15], some studies suggest that vaccinating pre-adolescents (<10 years) and adolescents (10–19 years) girls can be a public health challenge [16,17]. The HPV vaccine, which requires multiple doses delivered to pre-adolescent girls and targets a sexually transmitted infection (STI), therefore presents unique delivery challenges [16].

Despite the HPV vaccine's proven safety, efficacy and cost-effectiveness there exists a significant lag in its introduction in low- and middle-income countries (LMICs) [10]. In this study we examined the barriers and challenges to implementation of HPV vaccine in LMICs. As more countries consider its introduction, a review of the lessons from pilot projects and programme roll-outs in LMICs is essential to understand barriers to implementation and sustainability of an HPV vaccine programme.

## 2 Materials and methods

This paper uses two research methods, a literature review that preceded and informed subsequent key informant (KI) interviews. For the literature review four databases (PopLine, PubMed, Web of Science, Science Direct) were systematically searched with the following title search terms: “human papillomavirus” OR “HPV” AND “vaccine” OR “vaccination”. The database search was supplemented by a hand search of Google Scholar using the same title search terms. The search was restricted to English language items published since 2006, when the HPV vaccines became commercially available, until September 2012 [18]. The search yielded 5597 items, of which 2564 were duplicates, producing 3033 English language items published between 01/01/2006 and 30/08/2012 that were screened on title and abstract. Articles focusing solely on evidence from HICs were excluded. However, items that included a mix of HICs and LMICs were included in the thematic analysis. Country income groups were defined using the World Bank classification [9]. Items were included in the results if they reported on HPV vaccine implementation, including pilot projects, in LMICs. A total of 41 items were included in the thematic analysis (Table 2). Thematic analysis of the findings of the literature review preceded identifying broad areas under sociocultural, health system and political challenges as well as more specific issues including the sub-topics. These findings informed the design of the KII question guide. KIs explored themes that emerged from the literature review and represent a snapshot of the challenges and barriers during the vaccine implementation process.

A semi-structured interview guide was developed and KIs shared thoughts through open-ended questions on what they believed to be the greatest challenges to implementing the HPV vaccine in LMICs and provided perspective on the major themes and sub-sections that emerged from the literature review results. Formative research studies identified similar barriers to consider and overcome for the vaccine's introduction and supported the selection of the main themes within the literature review [11,19]. A draft interview guide was reviewed by individuals working in reproductive health at an international organisation to strengthen its face validity [20]. One of the authors (DW-J) was originally interviewed as a KI, prior to subsequent involvement in this paper, and did not influence selection of quotes for inclusion. The interview guides are available from the corresponding author. Ethical approval for this research was received from the London School of Economics, and informed consent was obtained from each KI after the nature and possible consequences of the research were explained. KIs gave written consent for anonymous quotes to be included in this paper. Seven interviews were conducted by the corresponding author (July 2011) using audio conference, each lasting between 20 and 50 min. We used the Internet to identify and contact key individuals working in the vaccine field as identified in key reports and journal articles. Four of the KIs were purposively selected for their research, policy or practical experience within HPV vaccines. Fifteen individuals were contacted from a range of non-governmental and international organisations and those implementing national HPV vaccine programmes and demonstration projects. Four refused and four did not respond to the request. KIs were not compensated for their participation. Characteristics of the KIs are described broadly in Table 1 in order to preserve their anonymity. Interviews were conducted in English, digitally recorded with permission, transcribed verbatim and anonymised. Interviews were analysed individually to identify overarching themes and were manually coded to assess each interview for topics and dissonance between informants [21].

## 3 Results

Items identified by the search included studies and experiences from individual countries (Peru, Vietnam, Uganda, India, Rwanda, Ghana, Tanzania, Malaysia, Indonesia, Kenya, Bhutan, Bolivia, Cambodia, Haiti, Lesotho and Nepal) and broad world regions. Three key barriers to the implementation of HPV vaccination in LMICs were identified in the literature review: sociocultural, health system and political (Table 2) and were supported by KIs. The sustainability of HPV vaccine programmes was identified as a cross-cutting challenge in the literature.

## 4 Sociocultural barriers

Sociocultural barriers to implementing the HPV vaccine identified in the literature are outlined in Table 2. Six of seven KIs highlighted potential issues to influence uptake and coverage of vaccine including: lack of knowledge amongst parents, cultural traditions and rumours. Communicating on the vaccine's preventive purpose and the need to provide adequate sensitisation at all levels were highlighted by both literature and KIs as important to overcome sociocultural barriers.

Despite theories that LMIC communities might express concerns about targeting only female adolescents to prevent an STI and the potential for social stigma, studies and KIs found that these concerns were rare [26,38,40–42].

“*Amazingly, I don’t think that there were as many barriers as we were expecting at the beginning*.” (KI-G).

“*There's going to need to be a very broad sensitisation plan that involves lots of different potential parties including the stakeholders but also groups, like religious groups etc*.” (KI-E).

Parental concerns within the literature focused on fear of future (in)fertility, increased and/or earlier sexual activity and the safety of the vaccine [23,24,26,27,48,58]. In Kenya, although baseline knowledge of cervical cancer, screening and the HPV vaccine were low, following information and communication about vaccine benefits, 95% of mothers reported that they would vaccinate their daughters [22]. Similar findings were reported in Ghana, Tanzania and Indonesia [23–25]. Several studies from LMICs report on the intention to vaccinate, rather than actual acceptance of vaccine [23,40–42]. However, recent experiences from vaccination demonstration projects and the national roll-out of HPV vaccine in Rwanda have reported success in reaching their primary target group with high coverage for all three doses and overcoming concerns regarding vaccinating adolescents against an STI [40–42,45,57,58].

The need for information about the dual preventive capacity of the HPV vaccine as both an STI and cervical cancer vaccine was viewed as essential to mitigating sociocultural issues by one KI.

“*There is a debate on whether it should be introduced as an STI vaccine or a cancer vaccine…and sometimes you hear that it's easier to say that it is a cancer vaccine because it doesn’t face as much taboo or debate, as does sexual health issues…Ideally it could be presented as a cancer vaccine, and little by little, it should be explained to the community but mainly to the target population that the vaccines protect against an infection due to HPV and that it is an STI*” (KI-G).

Timing of introducing the vaccine was identified by three KIs as important in order to avoid launching before conducting adequate community sensitisation, which could result in increased misinformation and low uptake. Education and sensitisation at all levels was essential to increase knowledge about cervical cancer and HPV, to mitigate sociocultural barriers was outlined in several studies [26,31,36,39–42,60] and by six KIs

## 5 Health system barriers

Insufficient infrastructure and human resources [28–31], financing, donation programmes and the delivery method were identified as significant health system barriers both within the literature and by all seven KIs.

### 5.1 Infrastructure & human resources

All seven KIs identified vaccine cold storage and one KI outlined the number of dosages as challenges to implementing the HPV vaccine in LMICs:

*“If we could have the vaccine without needing the cold chain, that would be great. So reducing doses and reducing our reliance on the cold chain, those are the general directions we would like to see it go*” (KI-F)

Logistical issues at the health provider level in particular for transporting staff and vaccines were obstacles, in part because of the large quantities of vaccine vials needed were identified by three KIs:

“*In terms of the vaccine itself…it's a single vial vaccine, and it takes up a lot of space*” (KI-E).

Insufficient human resources and capacity of staff were also reported as a challenge to vaccine delivery by three KIs:

“*Even before introducing this HPV vaccine we had limited health workers, especially those that are working in reproductive health clinics…adding HPV on top of all they are doing, they are already overwhelmed*.” (KI-C).

“*Adding the HPV vaccine…is a little bit complicated vaccine as compared to other vaccines because most other vaccines are done at the health care facility, but this one, the nurse, the health worker has to travel to the schools*” (KI-C).

### 5.2 Financing mechanisms

Financing for the HPV vaccine was reported as a significant challenge by all seven KIs and within literature [23,28,33,34,43,48,62]. The vaccine cost ranges from US$13 to more than US$100 for each dose to the public sector, making this unaffordable for many LMICs [45]. Progress has, however, been made through financing mechanisms, such as the GAVI Alliance (formerly known as the Global Alliance for Vaccines and Immunisation) or the Pan American Health Organization (PAHO) Revolving Fund for Latin America and Caribbean countries [35]. The GAVI Alliance aims to improve access of eligible LICs to vaccines through negotiating lower vaccine prices and co-financing, until countries can afford the vaccines [44]. In June 2011, Merck and Co., announced that vaccines will be provided to GAVI Alliance for $5 USD per dose [46,47]. The GAVI Alliance has now opened the window for eligible countries to receive support to implement demonstration projects or to support national programmes for countries that already have experience with delivering the vaccine. However, countries including many LMICs with a gross national income of more than $1550 per capita are not eligible for GAVI support [45].

Although increased priority and decreased price represent progress, reaching the large female adolescent population for three dosages is expensive. Five KIs identified other costs to start-up and sustain programmes as additional issues:

“*GAVI's announcement is not a panacea, it is one component of the financial barriers that then get broken down but these other components related to funding start-up costs and some onward implementation costs could be a potential barrier for countries*” (KI-A).

“*The initial cost is very challenging…I’m not talking about the cost to buy the HPV vaccine…but the cost to take care of the necessary logistics*.” (KI-C).

### 5.3 Donation programmes

Programmes such as the GARDASIL Access Program (GAP), implemented through Axios Healthcare Development with vaccine donated by Merck, were established to donate limited amounts of the vaccine to countries for demonstration projects to test HPV vaccine delivery strategies [36,51]. Such pilot programmes suggest that in-country ownership and capacity development may contribute to long-term success and sustainability of vaccine delivery [40–42]. Four KIs discussed donation programmes and challenges to sustaining efforts long-term.

“*There's the perfect trifecta for sustainability. You need money, you need political will and you need capacity. And until countries get fully supported on all three of those it's difficult for any program to be sustainable over time, and everybody has a role to play in that*” (KI-A).

Larger vaccine donations by GAP and the Merck Qiagen Initiative has also permitted several LICs to start national roll-out of HPV vaccine, including Rwanda [52,54]. Two KIs highlighted the issue of sustainability after the conclusion of these large-scale donation programmes:

“*It's what's going to happen at the end of that, that is critical, because by that point the whole population has been through this intervention, probably over a couple of years*” (KI-E).

“*You don’t want to see [countries] put in time, energy and effort into something that can’t continue, and it's disruptive to the other services if it's not well-planned*” (KI-F).

### 5.4 Delivery methods

Reaching adolescent girls to deliver vaccines and other health interventions was reported as a significant challenge. Demonstration projects and national programmes of HPV vaccine delivery found that several vaccine delivery methods including school-based programmes, at health-centres and the use of campaign approaches, were successfully employed to achieve coverage higher than that of many HICs, such as the United States, Denmark or the Netherlands [30,52,57,58,60].

For school-based vaccine programmes, the two KIs working in a LIC said poor timetable planning and documentation were concerns as these could result in missed dosages, decreasing vaccine coverage and overall programme success:

“*It's not just a one-off visit once a year, they’ve got to get in three days just within the school calendar year*” (KI-E).

“*It is a big challenge to adequately and effectively document the accepted names and the numbers of girls who received dose one, who received dose two and who received dose three*” (KI-C).

School absenteeism was the primary reason for not being vaccinated in programmes in India, Peru and Uganda and in government schools in Tanzania, although school attendance was reported to be very high in all countries, suggesting school-based methods are appropriate but measures are needed to capture pupils absent on the day of vacccination [57,58].

Inadequate school-based health programmes was highlighted by one KI as a potential threat to this delivery strategy [61]:

“*There is in theory a school-based health program but in practice it functions rather piecemeal. In practice some of those aspects of the school health program have been very fragmented, so the tetanus immunisation that is supposed to happen to girls aged about 15 in schools, basically often doesn’t take place*” (KI-E).

Two KIs suggested programmes might offer opportunities for other age-relevant services such as de-worming or nutritional supplements. This integrated approach is supported by the GAVI Alliance for countries applying for GAVI Alliance support and represents an opportunity to reduce the cost and burden on health systems of delivering separate interventions [45].

Out-of-school girls were reported in the literature review as a challenge, especially in countries with lower school enrolment and attendance [26,30,31,38,40,42,57,58].

“*Many women who are pregnant do bring their child once to some sort of clinic, and they get sort of captured, for infant stuff, but you know setting-up and sustaining school-based and out-of-school youth vaccination programmes is quite complex and…I don’t think one size will fit all here*” (KI-E).

Introduction of the HPV vaccine in India assessed the ability to reach this cohort through existing routine immunisation programmes or special campaigns (e.g. polio vaccines), and found a campaign approach at fixed times achieved high coverage [58]. The use of school-based programmes in combination with existing child public health days was used in Uganda to reach out-of-school girls, achieved 52.6% coverage, although the use of age as an eligibility criteria was found to decrease coverage as opposed to the delivery method [42,50,63]. Vietnam tested two delivery strategies, the school-based strategy achieved 83.4% in its first year and 93.4% in its second year, while the second strategy using community health centres achieved high coverage of 92.8% in its first year and 98.00% in its second, for all three doses [31]. In Rwanda, community involvement to identify girls who were either absent from, or not enrolled in school, combined with a national sensitisation campaign prior to delivery of the first dose, achieved over 93% coverage for all rounds of vaccination [52]. GAP in seven countries, including Bhutan, Bolivia, Cambodia, Haiti, Lesotho and Nepal, achieved average coverage of 88% for all three doses using school, health facility and mixed methods of delivery [60].

## 6 Political barriers

The literature and KIs identified a number of themes related to the political barriers and facilitators focusing on political will, coordinating stakeholders and competing health issues.

### 6.1 Political will

Lack of political commitment to new health technologies was identified as an important challenge to successful implementation of HPV vaccine programmes [28,32]. Expensive, new public health interventions, such as the HPV vaccine, demand more cost-effectiveness and sustainability evidence in order to convince policymakers [33,34]. It has been reported that decisions on health priorities and the introduction of new vaccines should be made nationally, based on evidence reflecting country-specific burden [34]. However, in practice, it is feared that international priorities, pharmaceutical company donations or subsidised vaccination programmes, such as the GAVI Alliance, influence political commitment and decision-makers to embark on HPV vaccination programmes [35]. Two KIs also highlighted the challenging decision-making process and the role of political priorities versus making decisions based on country-specific evidence.

“*We should be allowing countries to make a selection [of what to prioritise] and GAVI should reflect that. That prioritization within country plans and countries should be making decisions about [whether to fund] vaccines versus other non-health opportunities*” (KI-B).

“*In [country], we know it was the President, it was an election year, and he decided or had been persuaded that this was a good thing to do for [country] girls and women*” (KI-F).

“*I think that HPV unfortunately has taken an approach where it's played to some of the political needs…grasped by some of the political issues associated with HPV and risen to prominence. It may not be driven by evidence…it may be more driven by political priorities and the interests of a few champions*” (KI-B).

### 6.2 Involvement of stakeholders

The diverse group of stakeholders, including sexual and reproductive health, adolescent health, immunisation and cancer control groups, is unique to HPV [13]. Coordinating different stakeholders, each with their own priorities, was seen as an impediment to evidence-based decision-making and could also lead to competition of resources [35,43].

“*What's most important for sustainability is ensuring that everybody that has a role to play in that, plays the role that they’re best at and provides the support that's needed, especially for the countries that need it the most*” (KI-A).

Three KIs recognised the importance of stakeholder knowledge and approval.

“*We have to involve all the subgroups of the community because they all have to understand, they all have to agree*” (KI-C).

The HPV vaccine has progressed relatively rapidly through research and development and commercial availability [49]. Four KIs, primarily from international perspectives, attributed this success to cervical cancer advocacy groups, and suggested that advocacy will continue to play a key role in influencing implementation and policymakers:

“*I think cervical cancer advocacy has been quite good…they’ve been able to mobilize themselves quite effectively and the vaccine certainly has moved through its systems of development quite fast*” (KI-E).

### 6.3 Competing health priorities

Recognising HPV as a worthy cause of a country's limited resources, in comparison to other interventions, is one of the most important barriers to implementation and sustainability within the literature that must be conquered and was highlighted within much of the literature [29,38] and by all KIs.

“*It's hard to get decision-makers to prioritise an intervention that is so costly, when they have others, such as trying to achieve the MDGs, for under-fives and so on. Where do you put that money when you don’t have enough of it anyway*?” (KI-F).

“*It's a women's disease, it's sexually transmitted – that's a hard sell for some cultures*” (KI-F).

“*It's not a question of can we implement HPV vaccines and should it be a public health priority, but that we would be remiss if we didn’t make it a public health priority, we would be remiss in our obligations to those countries and those communities…now that we know that something works, you really do have a moral obligation*…” (KI-A).

Evaluation of vaccine priorities need to examine not only cost-effectiveness but also the affordability and distributional equity in country-specific settings. Weighing the benefits, costs and cost-effectiveness of the HPV vaccine to other interventions, such as the rotavirus vaccine, found similar number of lives saved over each target populations’ lifetime, despite targeting of different populations [55]. Two KIs felt that the prioritisation of the HPV vaccine is a challenge as a result of its delayed impact:

“*It would be very difficult to compare say a measles vaccine against an HPV vaccine, where you see the immediate effect of the measles vaccination, but your investment in HPV is much deferred*” (KI-F).

“*It kind of depends on whether you want to save lives right now or save lives in the future*” (KI-D).

LMICs that have not yet introduced comprehensive cervical cancer prevention strategies will have to consider whether it is feasible to invest in both screening and vaccination [56]. One KI reported establishing a comprehensive cervical cancer approach,

“…*was more challenging than we originally, perhaps naively thought, because you’re working with different programmes, you’re working with different target groups, and so while I think overall the introduction of the vaccine allows an opportunity to have a look at what your cervical cancer programme could consist of, it's difficult to roll those things out, the sequencing does not have to be simultaneous*” (KI-F).

## 7 Discussion

This study combines qualitative research with a review of current literature on the challenges and prospects to implementing the HPV vaccine in low- and middle-income countries. This research helps to document and share early experiences of the HPV vaccine in LMICs to enable countries, considering introducing national or demonstration programmes to build upon these lessons and establish best practices. The impact of timely implementation of the HPV vaccine within LMIC will be substantial, as every five-year delay in roll-out contributes to 2 million cervical cancer deaths [32].

This study has several limitations. The literature review only included items with English abstracts, so potentially relevant research from non-Anglophone settings were excluded. The small number of KIs may limit the generalisability of findings, however varying in-country and international perspectives were represented. The rapid change in the field of HPV vaccine policies and programmes means that this study represents only a snapshot in time of the early experiences and that continued progress has likely been achieved since conducting interviews. Although the majority of KIs were not part of the country immunisation programmes, three of the seven KIs worked directly with health providers and national immunisation teams through demonstration and pilot studies shaping future national strategies and programme decisions.

Finally, we present our findings from LICs and MICs together because of the relatively small number of studies and KIs for each country income group, and because of the cross-cutting themes that emerged from our thematic analysis. Early research from HICs and theoretical acceptance studies in LMICs predicted sociocultural issues as the most significant barrier for vaccine programmes, although targeted sensitisation programmes have been successful to overcoming this challenge and to achieving high vaccine acceptability and coverage rates in several LMIC. Our study identified health system challenges and political issues in LMICs as areas that represent the most significant challenges.

Health system barriers may undermine the ability of some LMICs to effectively implement high-quality HPV vaccine programmes. Securing sustainable financing for the vaccine including health system costs and maintaining donation programmes, represents a significant obstacle. A record low price for as little as US $4.50 per dose for LICs compared to more than $100 in HICs, was announced in May 2013, by the GAVI Alliance [64]. This reduced price and support system of co-payments by GAVI Alliance will further increase the affordability for LICs, however many MICs may still experience obstacles to funding and sustaining HPV vaccination programmes.

The HPV vaccine has been described as a unique intervention compared to other routine vaccination programmes, due to the multifaceted barriers experienced. In particular, administering three doses to girls 9–13 years, a new target group not previously reached by routine immunisations in most countries highlights obvious infrastructure and cold chain challenges. Reaching this cohort through special campaigns and school-based delivery methods may not be financially feasible or sustainable considering the additional and on-going costs to current immunisation structures [65–67]. This vaccine also raises novel political issues, in particular the coordination of multiple stakeholder groups. Robust local evidence and education about cervical cancer will be essential to garnering the necessary political support to roll-out vaccination.

Currently neither HPV vaccine has been licensed for girls less than 9 years of age, following the WHO recommendations target age range [18]. Continued research is needed on the period of immunogenicity provided by the vaccine and the possibility of reducing the number of doses without affecting vaccine efficacy [68].

Considerable progress has been made in several LMIC and it is important to understand lessons that are transferable to other settings. School-based programmes have been successful but alternative and innovative sensitisation and delivery methods will be important for achieving more equitable delivery. Demonstration and pilot projects in several LMICs have shown that methods including delivery through schools, special campaign, health centres or a multiple methods can reach a large proportion of eligible girls and that advocacy and sensitisation is essential.

GAP countries including Cameroon, Lesotho and Uganda have recently shared lessons from pilot activities including the importance of garnering political commitment, mobilising resources and recommending integrating the vaccine within existing immunisation structures for sustainability [69]. An evaluation of eight GAP pilots found that delivery through mixed-models in both schools and health facilities as most effective [60]. However, the authors acknowledge, that implementation of HPV vaccination needs to be adapted to local priorities, and that successful demonstration projects do not equate to a one-size-fits all for other LMICs. The results of HPV vaccine experiences so far in LMIC are encouraging and continued sharing of evidence, successes and challenges is critical to the replicability of successful HPV vaccine interventions worldwide.

## Figures and Tables

**Table 1:** Characteristics of key informants.

Key informant	Type of organisation	Position held	Region of expertise
A	International NGO	Programme Manager	International; works in LIC and LMIC (Africa and Asia)
B	International funding agency	Deputy Director	International
C	Local, country-based vaccination programme	HPV Project Coordinator	Sub-Saharan Africa; LIC
D	International funding agency	Senior Programme Officer	International
E	Local country-based vaccination programme	Epidemiologist	Sub-Saharan Africa; LIC
F	International organisation	Technical Officer	International
G	International organisation	Medical Officer	International

**Table 2:** Summary of challenges identified in literature.

Sociocultural	Health system & logistical	Political
Low knowledge of HPV and its relation to cervical cancer [22–27]	Infrastructure and human resources [28–31]	Lack of political will [28,32–37]
Societal values and stigma [38–42]	Financing Mechanisms and Vaccine Cost [23,28,33,35,36,39,43–48]	Involvement and coordination of diverse stakeholders [13,35,43,49]
Parental concerns of side effects including (in)fertility, early sexual onset, increased sexual activity and vaccine safety [23,24,26,27,31,48,50]	Donation Programmes [36,40–42,51–54]	Competing health priorities and evidence-based decision-making [29,32–35,37–39,55,56]
Vaccine target age and group [26,29,42,57]	Reaching out-of-school girls [26,30,31,38,40,42,57,58]	
Community sensitisation and advocacy [26,31,36,39,41,42,59,60]	Logistics and timetabling [28,60]	
	Delivery Strategies [30,31,38,40–42,57–61]	

## References

[1] WHO/ICO (2010). Human papillomavirus and related cancers in world. Summary report 2010.

[2] Kane M., Serrano B., de Sanjose S., Wittet S. (2012). Implementation of human papillomavirus immunization in the developing world. Vaccine.

[3] Forman D., de Martel C., Lacey C.J., Soerjomataram I., Lortet-Tieulent J., Bruni L. (2012). Global burden of human papillomavirus and related diseases. Vaccine.

[4] Adams M., Jasani B., Fiander A. (2007). Human papilloma virus (HPV) prophylactic vaccination: challenges for public health and implications for screening. Vaccine.

[5] Franco E.L., Mahmud S.M., Tota J., Ferenczy A., Coutlée F. (2009). The expected impact of HPV vaccination on the accuracy of cervical cancer screening: the need for a paradigm change. Arch Med Res.

[6] WHO (2007). Human papillomavirus and HPV vaccines: technical information for policymakers and health professionals.

[7] Onon T.S. (2011). History of human papillomavirus, warts and cancer: what do we know today?. Best Pract Res Clin Obstet Gynaecol.

[8] Garland S.M., Smith J.S. (2010). Human papillomavirus vaccines: current status and future prospects. Drugs.

[9] (2012). World Bank list of economies. 1 July 2012–1July 2013.

[10] Cervical Cancer Action (2012). Progress in cervical cancer prevention: the CCA report card.

[11] PATH (2012). Conducting formative research for HPV vaccination program planning. Practical experience from PATH.

[12] WHO (2006). Preparing for the introduction of HPV vaccines: policy and programme guidance for countries.

[13] Kane M.A., Sherris J., Coursaget P., Aguado T., Cutts F. (2006). Chapter 15: HPV vaccine use in the developing world. Vaccine.

[14] Kane M.A. (2010). Global implementation of human papillomavirus (HPV) vaccine: lessons from hepatitis B vaccine. Gynecol Oncol.

[15] Mackroth M.S., Irwin K., Vandelaer J., Hombach J., Eckert L.O. (2010). Immunizing school-age children and adolescents: experience from low- and middle-income countries. Vaccine.

[16] Pollack A.E., Balkin M., Edouard L., Cutts F., Broutet N. (2007). Ensuring access to HPV vaccines through integrated services: a reporductive health perspective. Bull World Health Organ.

[17] Brabin L., Greenberg D.P., Hessel L., Hyer R., Ivanoff B., Van Damme P. (2008). Current issues in adolescent immunization. Vaccine.

[18] WHO (2009). Human papillomavirus vaccines: WHO position paper. Biologicals.

[19] Bingham A., Janmohamed A., Bartolini R., Creed-Kanashiro H.M., Katahoire A.R., Khan I. (2009). An approach to formative research in HPV introduction planning in low-resource settings. Open Vaccine J.

[20] Green J., Browne J. (2005). Principles of social research. http://www.lavoisier.fr/livre/notice.asp?ouvrage=1266838.

[21] Saldana J. (2009). The Coding Manual for Qualitative Researchers. London.

[22] Becker-Dreps S., Otieno W.A., Brewer N.T., Agot K., Smith J.S. (2010). HPV vaccine acceptability among Kenyan women. Vaccine.

[23] Coleman M.A., Levison J., Sangi-Haghpeykar H. (2011). HPV vaccine acceptability in Ghana, West Africa. Vaccine.

[24] Jaspers L., Budiningsih S., Wolterbeek R., Henderson F., Peters A. (2011). Parental acceptance of human papillomavirus (HPV) vaccination in Indonesia: a cross-sectional study. Vaccine.

[25] Remes P., Selestine V., Changalucha J., Ross D.A., Wight D., de Sanjose S. (2012). A qualitative study of HPV vaccine acceptability among health workers, teachers, parents, female pupils, and religious leaders in northwest Tanzania. Vaccine.

[26] Bingham A., Drake K., LaMontagne S. (2009). Sociocultural issues in the introduction of human papillomavirus vaccine in low-resource settings. Arch Pediatr Adolesc Med.

[27] Madhivanan P., Krupp K., Yashodha M.N., Marlow L., Klausner J.D., Reingold A.L. (2009). Attitudes toward HPV vaccination among parents of adolescent girls in Mysore, India. Vaccine.

[28] Biellik R., Levin C., Mugisha E., LaMontagne D.S., Bingham A., Kaipilyawar S. (2009). Health systems and immunization financing for human papillomavirus vaccine introduction in low-resource settings. Vaccine.

[29] Louie K.S., de Sanjose S., Mayaud P. (2009). Epidemiology and prevention of human papillomavirus and cervical cancer in sub-Saharan Africa: a comprehensive review. Trop Med Int Health.

[30] Penny M., Bartolini R., Mosqueria N., LaMontagne S., Mendoza M.A., Ramos I. (2011). Strategies to vaccinate against cancer of the cervix: feasibility of a school-based HPV vaccination program in Peru. Vaccine.

[31] Epidemiology PaVNIoHa (2010). Evaluating HPV vaccine delivery strategies in Vietnam.

[32] Agosti J.M., Goldie S.J. (2007). Introducing HPV vaccine in developing countries – key challenges and issues. N Engl J Med.

[33] Goldie S.J., O'Shea M., Diaz M., Kim S-Y. (2008). Benefits, cost requirements and cost-effectiveness of the HPV16,18 vaccine for cervical cancer prevention in developing countries: policy implications. Reprod Health Matters.

[34] Andrus J.K., Toscano C.M., Lewis M., Oliveiria L., Ropero A.M., Davila M. (2007). A model for enhancing evidence-based capacity to make informed policy decisions on the introduction of new vaccines in the Americas: PAHO's ProVac initiative. Public Health Rep.

[35] Andrus J.K., Sherris J., Fitzsimmons J.W., Kane M.A., Aguado M.T. (2008). Introduction of human papillomavirus vaccines into developing countries – international strategies for funding and procurement. Vaccine.

[36] Winkler J.L., Wittet S., Bartolini R.M., Creed-Kanashiro H.M., Lazcano-Ponce E., Lewis-Bell K. (2008). Determinants of human papillomavirus vaccine acceptability in Latin America and the Caribbean. Vaccine.

[37] Munoz N., Franco E.L., Herrero R., Andrus J.K., de Quadros C., Goldie S.J. (2008). Recommendations for cervical cancer prevention in Latin America and the Caribbean. Vaccine.

[38] Tsu V.D. (2009). Overcoming barriers and ensuring access to HPV vaccines in low-income countries. Am J Law Med.

[39] Woo Y.L., Omar S.Z. (2011). Human papillomavirus vaccination in the resourced and resource-constrained world. Best Pract Res Clin Obstet Gynaecol.

[40] PATH (2010). HPV vaccination in Latin America: lessons learned from a pilot program in Peru. HPV vaccines: evidence for impact.

[41] PATH (2010). Shaping a strategy to introduce HPV vaccines in India: formative research results from the HPV vaccines: evidence for impact project. HPV vaccines: evidence for impact.

[42] PATH (2011). HPV vaccination in Africa: lessons learned from a pilot program in Uganda. HPV vaccines: evidence for impact project.

[43] Sankaranarayanan R. (2009). HPV vaccination: the promise & problems. Indian J Med Res.

[44] Batson A., Meheus F., Brooke S. (2006). Chapter 26: innovative financing mechanisms to accelerate the introduction of HPV vaccines in developing countries. Vaccine.

[45] Types of Support & FAQ [http://www.gavialliance.org/support/apply/].

[bib0230] 46Gavi welcomes lower prices for life-saving vaccines.

[bib0235] 47Vaccine Investment Strategy.

[48] Sam I.C., Wong L.P., Rampal S., Leong Y.H., Pang C.F., Tai Y.T. (2009). Maternal acceptance of human papillomavirus vaccine in Malaysia. J Adolesc Health.

[49] Brotherton J., Fairley C., Garland S., Gertig D., Saville M. (2010). Closing editorial: processes, opportunities and challenges after introduction of human papillomavirus vaccine. Sex Health.

[50] LaMontagne D.S., Barge S., Le N.T., Mugisha E., Penny M.E., Gandhi S. (2011). Human papillomavirus vaccine delivery strategies that achieved high coverage in low- and middle-income countries. Bull World Health Organ.

[51] Gardasil Access Program [http://www.gardasilaccessprogram.org/].

[52] Binagwaho A., Wagner C.M., Gatera M., Karema C., Nuttd C.T., Ngaboa F. (2012). Achieving high coverage in Rwanda's national human papillomavirus vaccination programme. Bull World Health Organ.

[bib0265] 53Rwanda, Merck and QIAGEN Launch Africa's First Comprehensive Cervical Cancer Prevention Program Incorporating Both HPV Vaccination and HPV Testing.

[54] QIAGENcares Program [http://www.qiagen.com/About-Us/Who-We-Are/QIAGENcares/].

[55] Kim S., Sweet S., Chang J., Goldie S. (2011). Comparative evaluation of the potential impact of rotavirus versus HPV vaccination in GAVI-eligible countries: a prelimiary analysis focused on the relative disease burden. BMC Infect Dis.

[56] Garland S.M., Cuzick J., Domingo E.J., Goldie S.J., Kim Y.T., Konno R. (2008). Recommendations for cervical cancer prevention in Asia Pacific. Vaccine.

[57] Watson-Jones D., Baisley K., Ponsiano R., Lemme F., Remes P., Ross D. (2012). Human papillomavirus vaccination in Tanzanian schoolgirls: cluster-randomized trial comparing 2 vaccine-delivery strategies. J Infect Dis.

[58] LaMontagne D.S., Barge S., Le N.T., Mugisha E., Penny M.E., Gandhi S. (2011). Human papillomavirus vaccine delivery strategies that achieved high coverage in low- and middle-income countries. Bull World Health Organ.

[59] Jacob M., Mawar N., Menezes L., Kaipilyawar S., Gandhi S., Khan I. (2010). Assessing the environment for introduction of human papillomavirus vaccine in India. Open Vaccine J.

[60] Ladner J., Besson M.-H., Hampshire R., Tapert L., Chirenje K.M., Saba J. (2012). Assessment of eight HPV vaccination programs implemented in lowest income countries. BMC Public Health.

[61] Zimet G.D. (2009). Potential barriers to HPV immunization: from public health to personal choice. Am J Law Med.

[62] Adams M., Jasani B., Fiander A. (2007). Human papillomavirus (HPV) prophylactic vaccination: challenges for public health and implications for screening. Vaccine.

[63] Watson-Jones D., Baisley K., Ponsiano R., Lemme F., Remes P., Ross D. (2012). Human papillomavirus vaccination in Tanzanian schoolgirls: cluster-randomized trial comparing 2 vaccine-delivery strategies. J Infect Dis.

[64] Millions of girls in developing countries to be protected against cervical cancer thanks to new HPV vaccine deals [http://www.gavialliance.org/library/news/press-releases/2013/hpv-price-announcement/].

[65] WHO (2012). Report of the HPV vaccine delivery meeting: identifying needs for implementation & research. HPV vaccine delivery meeting: 2012.

[66] Hutubessy R., Levin A., Wang S., Winthrop M., Ally M., John T. (2012). A case study using the United Republic of Tanzania: costing nationwide HPV vaccine delivery using the WHO Cervical Cancer Prevention and Control Costing Tool. BMC Med.

[67] WHO (2012). WHO Cervical Cancer Prevention and Control Costing (C4P) Tool user guide.

[68] Garland S.M. (2009). Can cervical cancer be eradicated by prophylactic HPV vaccination? Challenges to vaccine implementation. Indian J Med Res.

[69] Gardasil Access Program (2011). Gardasil access program: newsletter.

